# From the Modeling of an Electrochemical YSZ-Based Gas Sensor Used in Electrolysis Mode

**DOI:** 10.3390/s24020658

**Published:** 2024-01-19

**Authors:** Riadh Lakhmi, Jean-Paul Viricelle, Rouba Alrammouz, Mathilde Rieu

**Affiliations:** Mines Saint-Etienne, Univ Lyon, CNRS, UMR 5307 LGF, Centre SPIN, F-42023 Saint-Etienne, France; viricelle@emse.fr (J.-P.V.); rouba.alrammouz@emse.fr (R.A.); rieu@emse.fr (M.R.)

**Keywords:** electrochemical sensors, modeling, electrolysis mode, oxidant/reductant pollutants

## Abstract

Electrochemical sensors have been used for many decades. However, the modeling of such sensors used in electrolysis mode is poorly documented, especially in the case of multiple gases’ parallel actions. These are of great interest since they constitute the first brick to bring information on the natures and concentrations of gaseous mixture compositions, thanks to gray box modeling of sensor arrays, for example. Based on Butler–Volmer’s equations, a model assuming parallel reactions at gold cathode has been introduced in this article and confronted with experimental results. The establishment of the model is based on the extraction of three variables: the charge transfer coefficient “α”, the reaction order γ, and the reaction constant rate k_0_. Tests performed without pollutants and with different concentrations of oxygen could be nicely fitted using the model. The influence of the polarization current on the three variables of the model has been evaluated, showing a clear influence on the constant rate and the reaction order. Moreover, increasing the polarization current enabled us to obtain selectivity for oxidant gases. Similarly, the effect of the oxygen concentration was evaluated. Results showed that, in this case, the charge transfer coefficients “α” obtained for oxidant gases are quite different from the ones obtained in the polarization current varying conditions. Therefore, the model will be interesting in situations where polarization current and oxygen content are not varied together. Variation of polarization current can be quite interesting to obtain increased information for multivariate analysis purposes in constant oxygen content situations. Additionally, other parameters have to be considered for applications in which the oxygen content is bound to change, such as exhaust gases or combustion.

## 1. Introduction

Air pollution has become a major societal issue. With the increased development of motorized vehicles, pollution and toxic gases are being released in alarming concentrations into the atmosphere with all known consequences [1,2]. With concerns about the impact of human activity on global warming, European legislation has started to impose more and more drastic emission limits through the European emission standards (the euro 6D norm is currently in effect). The standards concern both particulate matter and polluting gases emitted from motorized vehicles. In the near future, industries, particularly those using combustion furnaces, will also be impacted by European pollution standards. Controlling the concentration of emitted gases and particles has, therefore, become a major issue in the automotive industry, and new sensors responding to the requirements of the harsh environment present in exhausts have emerged. In response to those requirements, electrochemical sensors based on solid-state ceramic electrolytes are ideal candidates for these applications [3,4].

However, just like gas sensors based on other transducing principles (electrical, mechanical, colorimetric, and optical), a lack of selectivity compels the users of electrochemical sensors’ to find different methods to extract the analyte’s nature and concentration. One of the methods currently used consists of modifying the composition of the sensor. This can be achieved by adding a selective, sensitive layer that responds to one target gas [5,6,7,8] or by integrating a filter that will prevent access to the sensor’s reaction sites to certain gases, similar to the work reported by J.Gao et al. [9]. It should be noted that in the case of electrochemical sensors, the sensitive layers are the electrodes themselves. As a result, both anode and cathode can be tuned [10,11,12].

Another method used to achieve good selectivity consists of using sensor arrays. This technique may be used as an alternative to the first one. In this case, no modification of the sensor’s composition is made. However, signal treatment based on multivariate analysis enables the extraction of the analytes’ nature and concentration due to the increased size of data collected by the different sensors of the array [13,14]. These arrays may be composed of different sensors based on the same transducing principle (arrays of metal-oxide (MOX) sensors [15,16,17], arrays of electrochemical sensors [18,19]), or can group sensors with different transducing principles.

Unfortunately, none of the previously reported methods was able to achieve satisfying results. As a result, both techniques still need improvement to minimize gas identification errors and reach lower limits of detection. One potential improvement that can be made lies within the use of knowledge models describing the physical behavior of the sensor instead of the black box models currently used in multivariate analysis of sensors’ data.

Therefore, better knowledge of the electrochemical sensor’s working principle, especially when exposed to gas mixtures, is crucial. This implies the establishment of predicting analytical and/or digital models describing the sensor’s behavior under different operating conditions. For all sensors, transduction is based on the modification of an output electrical characteristic induced by a physicochemical change in the sensor’s surrounding environment. Different models describing electrochemical sensors have been reported in the literature. Their aim is to achieve selectivity, similar to work that has been reported for other transducing systems, such as cantilever-based sensors [20].

The Yttria-stabilized zirconia (YSZ)-based electrochemical sensor presented in this article is based on the operating principle of a Solid Oxide Fuel Cell (SOFC). Various models exist in the literature describing the operating principle of this type of cell. For example, in 2007, V.M. Janardhanan et al. [21] proposed a literature review of the basic approaches for the general modeling of an SOFC fuel cell. Analytical models are based on mass balances in which the electrochemical equations are introduced and on energy balances in which the reaction enthalpies are involved, too. Those analytical equations are aimed to be used in numerical simulations. Concerning the electrochemical model, mechanisms in which charge transfer is the limiting step are considered through Butler–Volmer equations. Finally, the maximum cell potential E_rev_ is then expressed according to the Nernst equation for H_2_ oxidation at the anode, considering that there is no limitation due to gaseous species transport and that anodic and cathodic overpotential is neglected compared to the electrolytic resistance.

In 2016, L. Barelli et al. [22] modeled the behavior of an SOFC power engine to be able to handle thermal stresses overcome by those by implementation of adapted temperature PID (Proportional–Integral–Derivative) regulation controlling the air flow at the cathode. Mass balances and thermal balance equations are also considered in this case. Concerning the electrochemical modeling, the cell output voltage is calculated considering the Nernst equation to which loss terms (ohmic and polarization) are added. The loss terms are evaluated by the difference between the experimental and Nernst ideal curves. For variation of output power load (tens of kW), the airflow at the cathode is controlled by simulation.

M. Li et al. [23] proposed, in 2023, an analytic MATLAB/Simulink model concerning an improved SOFC power generation system. In this last one, each module of the SOFC power supply is modeled (heat exchanger, combustion chamber, and gas transmission pipelines). The modeling of each module is based on the division of the module into nodes, which are spatial divisions along the gas flow. For each node, modeling is based on mass and energy balances. In the mass balances, reaction rates are expressed for H_2_O formation from H_2_ reaction at the anode and for O_2_ dissociation at the cathode, enabling the calculation of the flow rates relative to each gas. Fuel cell voltages are extracted from the energy balance equations and output currents.

In those three literature examples, SOFC models are developed and simplified according to their final use, which is, obviously, the “fuel cell mode” use, i.e., no external current or voltage is applied between electrodes. Overpotentials are, in this case, neglected because they are negligible compared to the potential linked to the resistance of the electrolyte.

As far as models for gas sensors are concerned, those found in the literature are dedicated to “fuel cell mode” use. In some cases, a diffusion layer is introduced on one of the electrodes, as is the case for Y. Dong et al. [24] who have developed an oxygen-selective electrochemical sensor using a (CuO)_0.1_(8YSZ)_0.9_ layer, the role of which is to make the diffusion step the limiting step. The sensor response (limiting diffusion current) is, then, based on the diffusion properties of oxygen through this layer. Concentrations between 0 and 4% oxygen were measured, and cross-sensitivities to H_2_O and CO_2_ were characterized.

Another successful model for describing the response of diffusion-free electrochemical sensors used in “fuel cell mode” is the mixed potential model. Indeed, in tests carried out on electrochemical sensors, when several species can react at the same time on the same electrode, the thermodynamic equilibrium reached on each of the electrodes is different from the Nernst equilibrium. In this case, a more suitable model, presented in review articles by S. Haley et al. [25] and T. Ritter et al. [26], is the theory of mixed potentials. This theory has been used, for example, by X. Hao et al. [27] to explain the response of the electrochemical sensor developed from a conventional YSZ electrolyte and a new type of measuring electrode: Nd_2_AO_4_ (with A=Cu, Ba and Ni) to concentrations of H_2_S below 1 ppm. Those kinds of mixed potential-based electrochemical sensors have already been used in several different applications. For example, E.L Brosha et al. [28] studied, for automotive exhaust gas applications, the response to C_3_H_6_ and CO of electrochemical sensors constituted of metal-oxide LMO sensing electrode (LaMnO_3_)/CGO electrolytes (Ce_0.8_Gd_0.2_O_1.9_)/Pt reference electrode. T. Liu et al. [29] also used CGO-based electrolyte sensors for diabetes diagnosis. Indeed, patients suffering from this disease will expire a breath containing a higher concentration of acetone that will be detected by the sensor.

In the model developed in this paper, electrolysis mode is used mainly with the aim of discriminating gases through a difference of behavior according to polarization currents. In this case, the effect of overpotentials becomes important, and overpotentials’ contribution to the sensor’s signal is no longer negligible. It even becomes the key element of the model. Moreover, a special mechanism is proposed in the developed model in which the signal is governed by parallel reactions occurring at the cathode.

This paper reports the analytical modeling of an electrochemical sensor. The sensor’s electrical response is linked to the electrochemical reaction occurring at the cathode and anode. Therefore, it will provide information on the analyte’s concentration and the redox behavior of the species involved in the reaction. The models proposed in this work describe the response of an electrochemical sensor used in electrolysis mode. In Section 2, the architecture of the electrochemical sensor used in this study is described in detail, and the proposed models are explained. The first one concerns the interaction of the sensor with O_2_ only, then the second concerns interactions with oxidant gases and O_2,_ while the third is dedicated to the effect of reducing gases together with O_2_ on the sensor’s signal. The models result from the association of the Butler–Volmer equations [30] and an equivalent electrical circuit. Section 3 compares these models to different experimental curves to test their robustness. For this purpose, a MATLAB (R2017b) code was developed to extract the models’ kinetic parameter. Section 4 concludes this article and highlights the advantages of the adopted approach for the selective detection of gases.

## 2. Materials and Methods

### 2.1. Sensor’s Physical and Electrochemical Description

**Figure 1:**
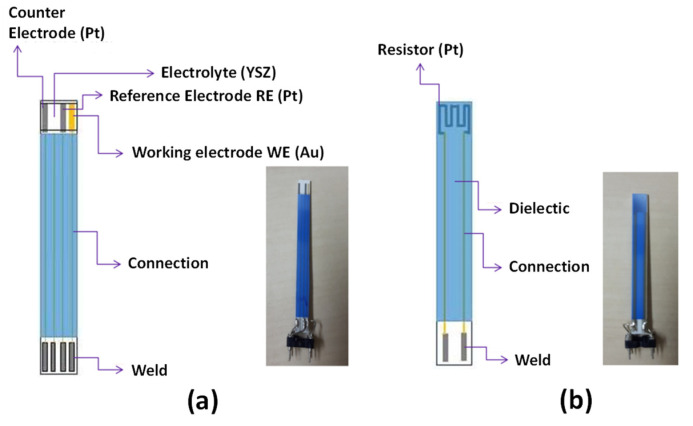
(**a**) Sensing side of the NOX sensor, (**b**) Heating side of the NOX sensor.

The considered system is an electrochemical planar sensor. On its “sensing side”, the sensor is composed of a YSZ layer screen-printed onto a 5 cm × 0.5 cm alumina substrate and three metallic electrodes screen-printed onto the YSZ layer (Figure 1). A platinum resistance on the “heating side” of the alumina substrate enables the heating of the YSZ layer by the Joule effect to a temperature at which the ionic conductivity of the solid electrolyte becomes reasonable. Additionally, the same platinum heater is used to monitor the temperature. On both sides, a dielectric layer (the blue color in Figure 1) guarantees electronic isolations of electrodes’ or resistance’s wirings. Low currents (25–150 nA) were applied between the working gold electrode (WE) negatively polarized as a cathode, and the platinum counter electrode (CE) playing an anode’s role. The sensor response (${\Delta V}_{ref}$) was measured as the potential difference between the reference platinum electrode (RE) and (WE). Several sensors were tested in this work. The sensitivity and selectivity of those sensors to Nitrogen Oxides (NOX) were investigated in a test bench in the temperature range 450–550 °C for atmospheres containing O_2_ (1–12 vol.%), H_2_O (1.5% absolute humidity) and N_2_. Alternatively, various polluting gas injections are performed: NO (0–1000 ppm), NO_2_ (0–1000 ppm), CO (0–1000 ppm), and NH_3_ (0–20 ppm). Analyte detection tests were conducted in a test bench developed in the laboratory. More details about the test bench and experimental facilities can be found in [31,32,33].

**Figure 2:**
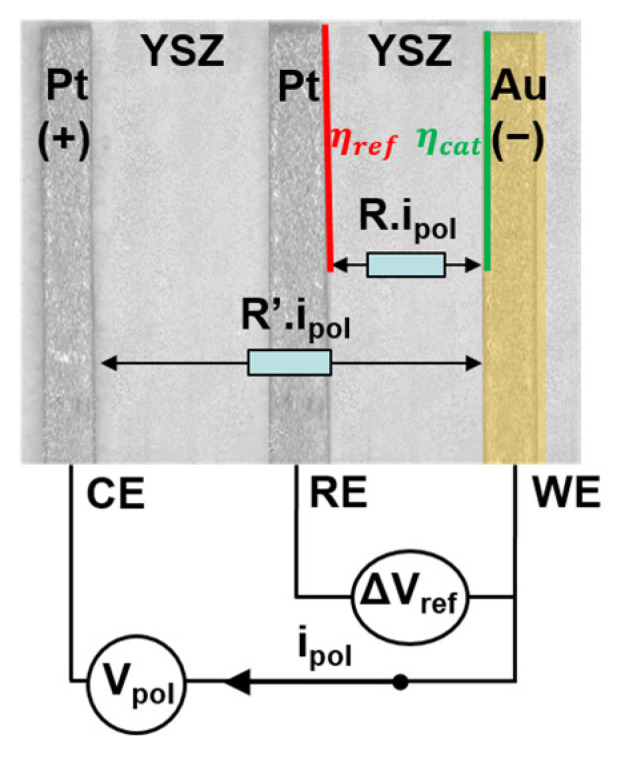
Measurement scheme of a sensor subjected to a polarization current.

As mentioned earlier, the sensor is operated in its electrolysis galvanostatic mode, i.e., a constant current I is applied between WE and CE and a potential difference ${\Delta V}_{ref}$ is measured between WE and RE: ${\Delta V}_{ref}=V_{RE}-V_{WE}$ (Figure 2). The measured voltage is given by the following equation [26]:

(1) $${\Delta V}_{ref}\approx R.I+{\Delta V}_{0}-\eta_{cat}$$

where ${\Delta V}_{0}$ is the output voltage measured between WE and RE when no current is applied between the electrodes ($\eta_{cat}$ is considered null at I = 0), $\eta_{cat}$ is the cathodic overpotential, and R is the electrolyte resistance. The overpotential is linked to an additional quantity of energy required (compared to the one expected thermodynamically) by a reaction to occur over an electrode. Therefore, it is closely linked to the reaction kinetics over the considered electrode. In our case, platinum, which is a well-known oxidation catalyst [34,35] is used as the anode. The consequence will be quite low overpotential $\eta_{ref}$ for the platinum anode compared to the gold cathode. Then, $\eta_{ref}$ will be neglected in this study. Extraction of the overpotential $\eta_{cat}$ requires that the determination of ${\Delta V}_{0}$ and R. ${\Delta V}_{0}$ is the measured ${\Delta V}_{ref}$ signal when the polarization current is null. It is measured for each gas concentration that will be used in electrolysis mode. R is obtained from the electrolyte impedance measurement at 100 Hz thanks to an electronic circuit developed at the laboratory.

The overpotential η has been, for us, of interest for many years [34] since it best reflects the effects of the gas on the sensors’ response. Since the sensor is of an electrochemical nature, the current flow is made possible by the redox reactions happening at both electrodes. It should be noted that the current has an ionic nature (O^2−^ ions) in the electrolyte and an electronic nature in the external circuit linking the electrodes. The transition between one form of current to the other is guaranteed by the redox reactions. At the triple phase boundaries, the current form is changed from ionic to electronic at the anode (Pt electrode here) and inversely at the cathode (Au electrode here). This change implies an energy supply, which can be electrically interpreted as a potential evolution. Therefore, the overpotential can be seen as the voltage across an interface resistance (the capacitive component of the interface, linked to a transient state of adsorption [36] and the double layer phenomena, which is unaddressed in this paper):

(2) $$\eta \left(I\right)=R_{interface}.I+\eta_{0}$$

$\eta_{0}$ is the overpotential value at I = 0 A($\eta_{0}$ is considered null, as mentioned earlier). The gaseous composition of the surrounding atmosphere will have a strong impact on the value of $R_{interface}$. Depending on the present gases, an evolution of the overpotential is experimentally observed. An increase in the absolute value of overpotential is noticed when a reducing gas is added to the atmosphere, and a decrease of this last one is observed when the sensor’s atmosphere is modified by the addition of an oxidizing gas.

The model developed in this work aims to offer a prediction $\eta_{mod}$ of the overpotential $\eta_{exp}$, experimentally determined. Many parameters like the temperature, the oxygen, and polluting gas concentrations or the imposed current were modulated to test the model’s robustness. From this, it will be deduced that the oxidizing analytes will have a “positive” action on the current flow (decrease of the resistance interface: $R_{interface}$), whereas reducing gases will have the opposite effect.

The global kinetics of a reaction relies on three phenomena, each guided by their own kinetics: the ionic or gaseous species transport to the electrodes, the molecules’ adsorption, and the charge transfer at triple phase boundaries (points of contact of electrolyte, electrode, and gaseous phase). For the polarization currents chosen in this work, the high oxygen concentration and the constant flow rate of 60 L/h are supposed to prevent kinetics limitation by diffusion transport. Even though including adsorption models would have enabled us to obtain information on the transitory phases (polarization current change) and possible drift occurring, we chose not to consider the adsorption kinetics models to limit the number of undetermined parameters. For these reasons, the physics included in the developed model to fit the experimental results are those considering kinetics limited by charge transfer.

In many kinetics approaches, reactions are considered elemental, and a first order is chosen for the kinetic study [37,38]. Sometimes, the reaction order can rely on the operational conditions (especially in the case of gases) and can be a function of temperature, for example. A proper evaluation of the reaction order requires exposing the sensor to different concentrations of the reactant gases while varying the experimental conditions, like the temperature, to check their effect on the reaction order. Then, the current (*I*) can be linearly linked to both the reductant and oxidant concentrations (by convention, the oxidation current is positive, and the reduction current is negative):

(3) $$I=nFS\left(k_{+}{C_{red}}^{\gamma }-k_{-}{C_{ox}}^{\gamma }\right)$$

where $k_{+}$ and $k_{-}$ are respectively the oxidation rate constant and the reduction rate constant, $C_{red}$ and $C_{ox}$ are respectively the reductant and oxidant concentration at the electrode/electrolyte interface, γ the reaction order, S the electrode surface, F the Faraday constant, and n the number of electrons exchanged during the redox reaction. It can be demonstrated that [39]:

(4) $$k_{+}=k_{0}.exp\left(\frac{\left(1-\alpha \right).nF.\left(E-E_{0}\right)}{RT}\right)$$

(5) $$k_{-}=k_{0}.exp\left(\frac{-\alpha .nF.\left(E-E_{0}\right)}{RT}\right)$$

where $k_{0}$ is the intrinsic standard rate constant, E the electrode potential, $E_{0}$ the standard potential of the redox couple involved (for example, E_0_ (O_2_/O^2−^) = 1.12 V vs. SHE), E the electrode potential and α the cathodic charge transfer coefficient. Here, we assume that the sum of cathodic and anodic charge transfer coefficients is equal to 1. As a result, (1−α) represents the anodic charge transfer coefficient.

When the global current I is null, the cathodic and anodic currents are equal. We define by current exchange: $I_{0}$ the value of this anodic or cathodic current from Equations (3)–(5):

(6) $$I_{0}=nFS.k_{0}.exp\left(\frac{\left(1-\alpha \right).nF.\left(E_{eq}-E_{0}\right)}{RT}\right).{C_{red}^{*}}^{\gamma }$$

(7) $$I_{0}=nFS.k_{0}.exp\left(\frac{-\alpha .nF.\left(E_{eq}-E_{0}\right)}{RT}\right).{C_{ox}^{*}}^{\gamma }$$

$C_{ox}^{*}$ and $C_{red}^{*}$ are respectively the gas concentration in the atmosphere close to the sensor and the reductant concentration inside the electrolyte (O^2−^ ion). Here, we suppose that the electric current is low enough and that the mass transfer by convection (gas flow) is fast enough so that the species concentration far from the electrode remains similar to the one near the electrode (diffusion is not the limiting step). This implies that $C_{ox}^{*}=C_{ox}^{\;}$ and $C_{red}^{*}=C_{red}^{\;}$.

$E_{eq}$ is the electrode potential at equilibrium that can be expressed by Nernst law:

(8) $$E_{eq}=E_{0}+\frac{R.T}{n.F}.ln\left(\frac{a_{ox}}{a_{red}}\right)$$

$a_{ox}^{\;}$ and $a_{red}^{\;}$ are respectively the activity of the gas in the atmosphere near the sensor (for the gaseous analytes) and the reductant activity of the adsorbed analyte (here the O^2−^ ions) considered equal to 1 since O^2−^ are present in the solid phase in the electrolyte matrix.

(9) $$a_{ox}=\frac{p_{ox}}{p{}^{\circ}}=x\frac{p_{tot}}{p{}^{\circ}}$$

where $p_{ox}$ is the partial pressure of the oxidant gas, p° the reference pressure (1 bar), $p_{tot}$ the total pressure of the gas mixture (in our case, it is the atmospheric pressure) and x the molar fraction of the oxidant gas. Since, in our case, the total pressure is the atmospheric pressure, which is about 1 bar, $a_{ox}$ is assimilated to x. Then, taking into account the expression of the overpotential: $\eta =E-E_{eq}$, and all equations previously mentioned, the current (*I*) can be given by:

(10) $$I=I_{0}.\left({\left(exp\left(\frac{nF\eta }{RT}\right)\right)}^{1-\alpha }-{\left(exp\left(\frac{nF\eta }{RT}\right)\right)}^{-\alpha }\right)$$

From this equation, approximations can be performed to extract overpotential:

- If $\left\|\eta \right\|$ is very low, the current value can be approximated by a first-order Taylor series expansion:
  (11) $$\eta \approx \frac{I.RT}{I_{0}.nF}$$
- If $\left\|\eta \right\|>100\;mV$ and η<0 (As it will be seen later, for our tested polarization currents and gaseous compositions, overpotential at gold cathode has been measured between −1.1 V and −0.1 V):
  (12) $$\eta \approx -\frac{RT}{\alpha nF}ln\left(\frac{\left|I\right|}{I_{0}}\right)$$

By association of Equations (7)–(12), the following expression can be formulated:

(13) $$\eta \approx -\frac{RT}{\alpha nF}ln\left(\frac{\left|I\right|}{nFS.k_{0}.exp\left(\frac{-\alpha .nF.\left(E_{eq}-E_{0}\right)}{RT}\right).{C_{ox}^{\;}}^{\gamma }}\right)$$

Used gases have been considered ideal gases. Then, using the Ideal Gas Law and Equation (8), the last expression can be reformulated by:

(14) $$\eta \approx -\frac{RT}{\alpha nF}ln\left(\frac{\left|I\right|.{\left(RT\right)}^{\gamma }}{nFS.k_{0}.x^{\gamma -\alpha }.{p_{atm}^{\;}}^{\gamma }}\right)$$

### 2.2. Electrochemical and Associated Electrical Models

According to the gaseous environment, different reactions will occur at the anode and the cathode. For the electrochemical model, we can distinguish three cases:

- Model 1: “Base gas” case

In the “base gas” case, the atmosphere around the sensor is composed of O_2_ (0.5–12 vol.%), H_2_O (1.0% absolute humidity) and N_2_. In this case, the reactions taking place are the following:

- at the cathode: O_2_ + 4e^−^ → 2O^2−^
- at the anode: 2O^2−^ → O_2_ + 4e^−^

**Figure 3:**
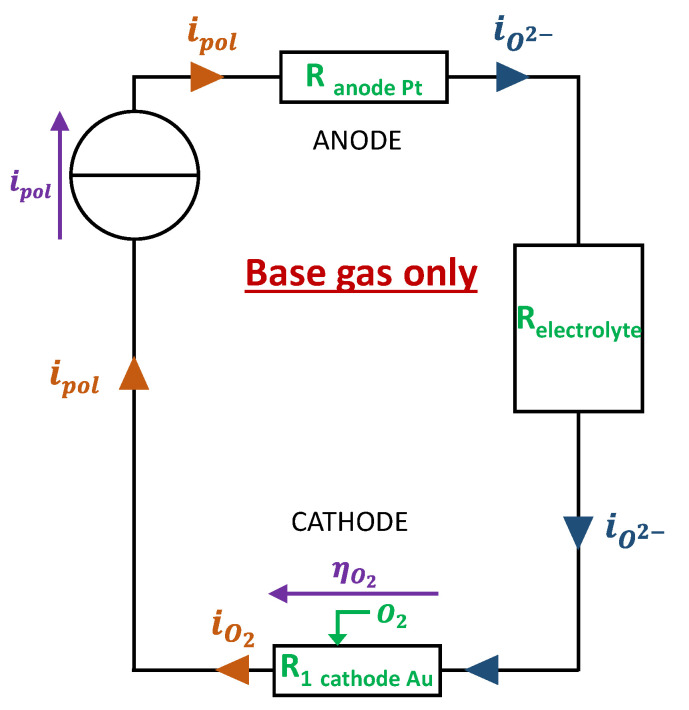
Electrical design of the electrode for model 1: base gas only.

The electrical modeling reliant on the electrochemical description is proposed in Figure 3. It should be noted that, in this last one, the “conventional direction of current” has been used to represent the current flows. In international standards, they are arbitrarily defined as the opposite direction in which electrons or anion (O^2−^) flow. Therefore, the direction of electrons goes from the generator to the cathode and the direction of O^2−^ anions goes from the cathode to the anode. Indeed, at the cathode, adsorbed O_2_ molecules are dissociated and, thanks to the electrons brought by the generator, are converted to O^2−^ ions transporting charges in an ionic form through the electrolyte to the anode.

The modeled overpotential under base gas can be expressed by the following equation:

(15) $$\eta_{mod}=-\frac{R.T}{\alpha_{base}.n_{1}.F}.ln\left(\frac{i_{pol}.{\left(R.T\right)}^{\gamma_{base}}}{S.k_{0_{base}}.n.F.{x(O_{2})}^{\gamma_{base}-\alpha_{base}}.{P_{atm}}^{\gamma_{base}}}\right)\;$$

where the electrode surface $S=3.68\;{mm}^{2}$, $a(O_{2})$ is the activity of dioxygen, n=4 is the number of electrons exchanged in the reduction reaction of O_2_. The modeled overpotential evolution according to time during exposure of the sensor to base gas (varying O_2_ concentration) will, in the following part, be fitted to the experimental overpotential curve thanks to $\alpha_{base}$, $k_{0_{base}}$, $\gamma_{base}$ parameters.

- Model 2: Presence of an oxidizing gas (NO_2_, NO)

When an oxidizing gas (NO_2_ or NO) is added to the “base gas”, the following reactions are expected:

- at the cathode: O_2_ + 4e^−^ → 2O^2−^

NO_2_ + 4e^−^ → ½ N_2_ + 2O^2−^

or

NO + 2e^−^ → ½ N_2_ + O^2−^

- at the cathode: 2O^2−^ → O_2_ + 4e^−^

**Figure 4:**
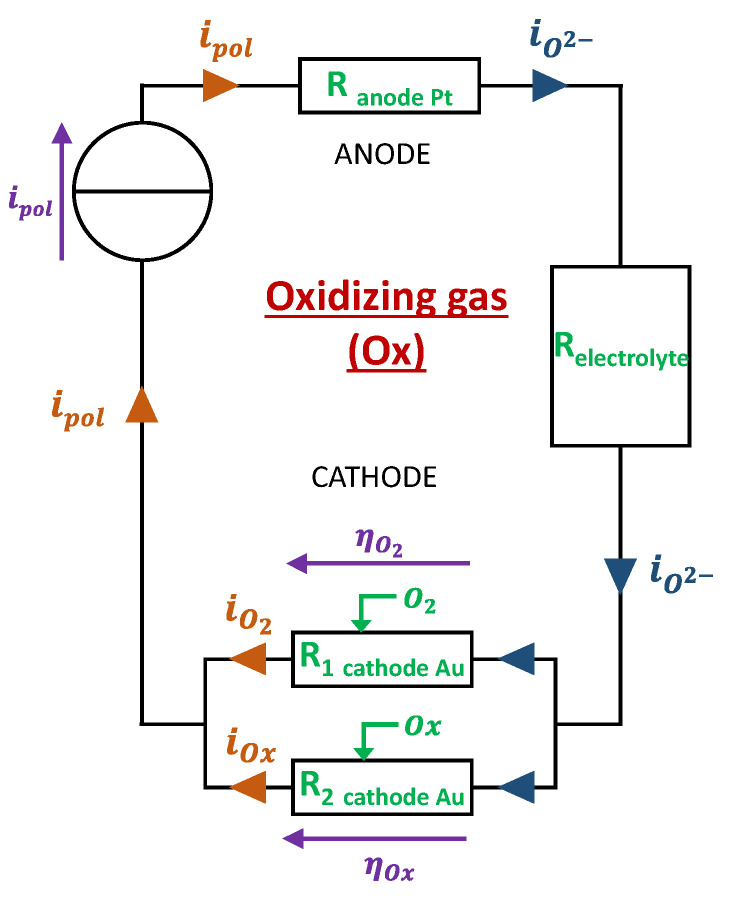
Electrical design of the electrode for model 2: addition of an oxidizing gas.

The current flow when an oxidizing gas is present is facilitated by the contribution of NO_2_ and NO, bringing more O^2−^ which are the current-carrying ions. The consequence is the reduction of the gold cathode/gas/electrolyte interface resistance and the reduction of the cathode’s overpotential. This can be electrically modeled by the scheme in Figure 4.

It can be deduced that, compared to the “base gas” case, when an oxidizing gas is present, the imposed polarization current $i_{pol}$ is sustained not only by the $O_{2}$ reaction at the interface but also by the polluting oxidant gas reduction: $i_{pol}=i_{O_{2}}+i_{Ox}$. Moreover, according to Kirchhoff law, the overpotentials $\eta_{O_{2}}$ and $\eta_{Ox}$ should be equal. Then, the problem is to determine the quantity of current that will be sustained by O_2_ and the quantity that will be sustained by the oxidant gas. This requires another equation linking $i_{O_{2}}$ and $i_{Ox}$ to a system with two equations and two unknowns.

A hypothesis that gave the better modeling results consists of considering that the ratio of current sustained respectively by O_2_ and the oxidant gas is the same as the ratio of exchange currents obtained when the global current is null:

(16) $$\frac{i_{O_{2}}}{i_{Ox}}=\frac{I_{0\;(O_{2})}}{I_{0\;(Ox)}}$$

$I_{0\;(O_{2})}$ and $I_{0\;(Ox)}$ can be calculated respectively according to $\alpha_{base}$, $k_{0_{base}}$, $\gamma_{base}$ (relative to reduction reaction of O_2_) and $\alpha_{gas}$, $k_{gas}$, $\gamma_{gas}$ (relative to the reduction reaction of oxidant gas).

Finally, when an oxidant is added to the base gas, cathodic overpotential is changed to the following value:

(17) $$\eta_{mod}=-\frac{R.T}{\alpha_{gas}.n_{1}.F}.ln\left(\frac{i_{Ox}.{\left(R.T\right)}^{\gamma_{gas}}}{S.k_{0_{gas}}.n.F.{a(O_{2})}^{\gamma_{gas}-\alpha_{gas}}.{P_{atm}}^{\gamma_{gas}}}\right)$$

where n is the number of electrons exchanged in the reduction reaction of the oxidant gas considered. The modeled overpotential evolution according to time will be fitted to the experimental overpotential curve in the following part, thanks to $\alpha_{base}$, $k_{0_{base}}$, $\gamma_{base}$ and $\alpha_{gas}$, $k_{0_{gas}}$, $\gamma_{gas}$ parameters. During exposure, the sensor will alternatively be exposed to base gas (varying O_2_ concentration or not) and oxidant gases (fixed concentrations or varying ones).

- Model 3: Presence of a reducing gas (NH_3_, CO)

**Figure 5:**
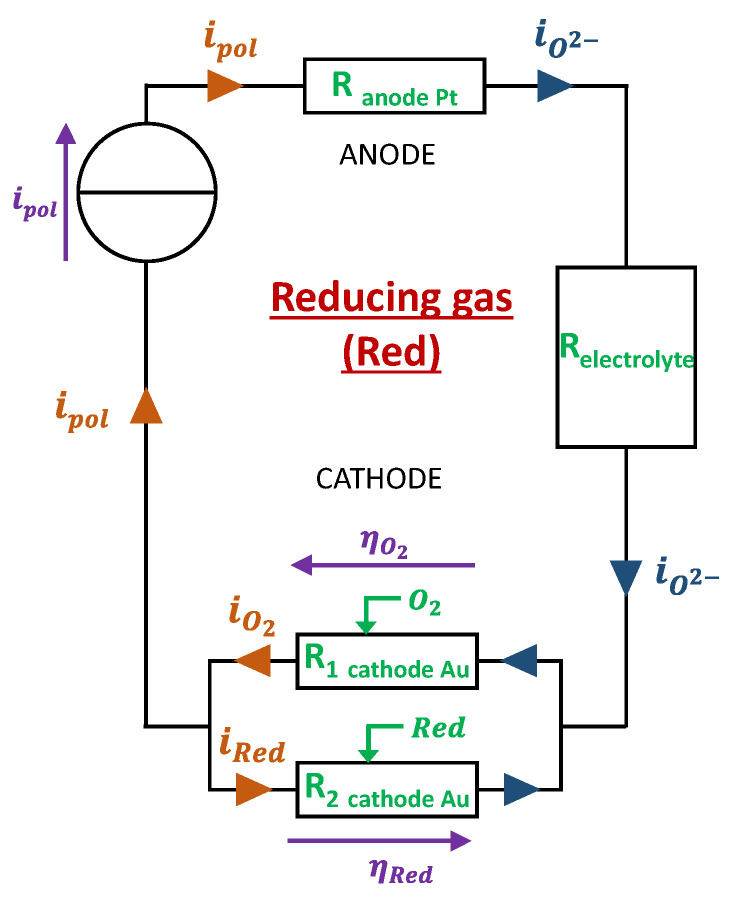
Electrical design of the electrode for model 3: addition of a reducing gas reacting with O^2−^.

When the sensor is exposed to oxidant gases in galvanostatic mode (constant $i_{pol}$) like it was in our case, the absolute value of the overpotential is seen to decrease. This is explained by the fact that the reduction of the oxidant gas decreases the interface resistance by providing O^2−^ ions as shown in Figure 5. Regarding exposure of the sensor to reducing gases, we experimentally observed a tendency of the overpotential to maintain constant or slight increases (in absolute value). To explain this behavior, we propose a mechanism in which the reducing gases will react with O^2−^ ions produced by the reduction of O_2_ at the cathode. The model associated with this reaction mechanism is described hereafter for two reducing gases (CO and NH_3_):

- at the cathode: O_2_ + 4e^−^ → 2O^2−^

CO + O^2−^ → CO_2_ + 2e^−^

or

NH_3_ + 5/2 O^2−^ → NO + 3/2 H_2_O + 5e^−^

- at the anode: ${2O}^{2-}\to O_{2}+4e^{-}$ 2O^2−^ → O_2_ + 4e^−^

This model assumes that the presence of CO or NH_3_ tends to decrease the quantity of *O*^2−^ ions. Thus, the current flow will be made more difficult due to the decrease in the number of available charge carriers. This will, therefore, increase the gold cathode/gas/electrolyte interface resistance and the cathode’s overpotential.

When a reducing gas is present, the imposed polarization current $i_{pol}$ is sustained only by the $O_{2}$ reaction at the interface. Moreover, a part of the current that comes from $O_{2}$ reduction reaction is used to oxidize the present reducing gas: $i_{pol}=i_{O_{2}}-i_{red}$. The consequence is the increase in the cathodic overpotential compared to the “base gas” case. Finally, the addition of a reductant to the base gas will change the cathodic overpotential in accordance with the expression (17). Nevertheless, in this case, the expression of $i_{Ox}$ will be replaced by: $i_{red}=i_{O_{2}}-i_{pol}$.

### 2.3. Multivariate Fitting Methods

**Figure 6:**
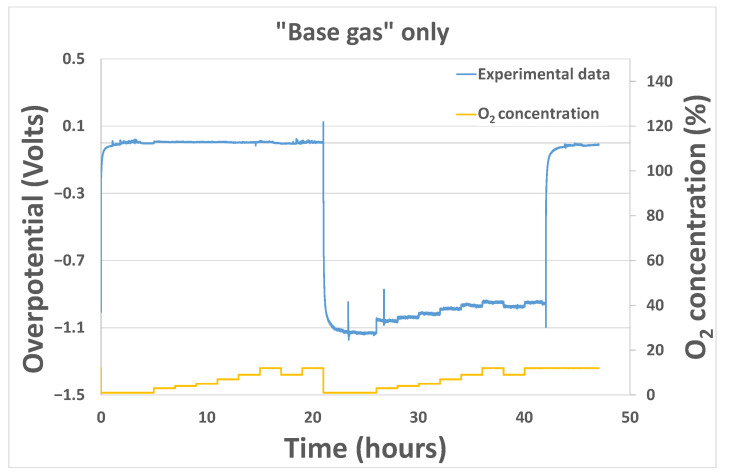
Example of response curve obtained experimentally under base gas modifying O_2_ concentration.

As mentioned previously, experimental sensor response curves were obtained by modifying analyte concentrations or polarization currents. From those raw response curves, experimental overpotential according to time could be extracted from Equation (1) (Figure 6).

MATLAB (2017b) algorithms were developed, based on the previously mentioned laws, in order to check if there are sets of parameters (α, γ, k_0_, …) allowing a good correspondence between the experimental and the modeled data. This corresponds to a three-parameter non-linear multivariate fitting problem. For convenience, vector gathering the three parameters was noted: $p=\left(\begin{array}{c} \alpha \\ \gamma \\ k_{0} \end{array}\right)$. Three methods were chosen and tested to solve this problem: the Least Squares method, the Newton–Gauss method, and the Levenberg–Marquardt method. For each one, an iterative algorithm was developed with MATLAB to extract the vector p values, allowing the lowest difference between the experimental and modeled data. Then, a comparison of their fitting performance was performed through the RMSE (Root Mean Square Error). Besides, during the implementation of the different methods, it was noticed that the Newton–Gauss and Levenberg–Marquardt methods were very sensitive to the initialization conditions. This is why, in the completed versions of the code, a fitting with the method of least squares was first performed. Then, the parameters obtained were used as input parameters for the two other methods. The values of the parameters displayed in this work are those of the method that gives the best fit, i.e., the smallest RMSE.

## 3. Results and Discussions

### 3.1. “Base Gas” Alone Case

**Figure 7:**
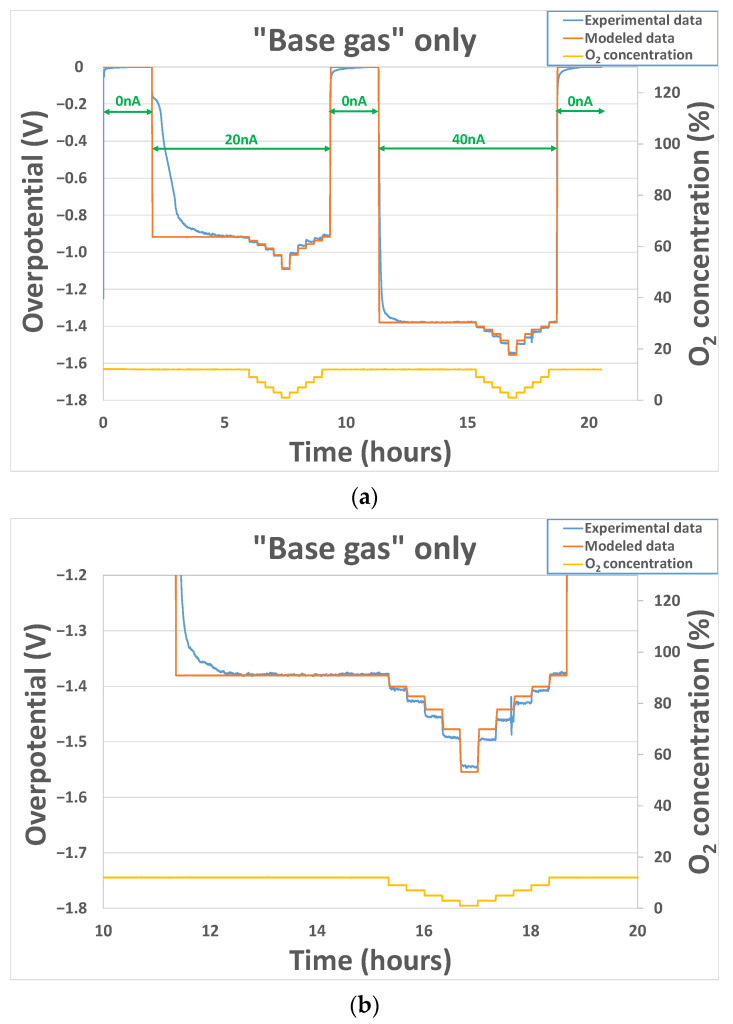
(**a**) Experimental and modeled overpotential evolution according to the gaseous environment (base gas–O_2_ variation from 1 to 12% at 500 °C) and polarization current sequence (0 nA–20 nA–0 nA–40 nA–0 nA) at 500 °C; (**b**) Zoomed view of the second part with polarization current of 40 nA.

Results, shown here, were obtained for two sensors heated at temperatures from 450 °C to 550 °C and exposed to base gas only, with different oxygen concentrations from 1% to 12%. The polarization cycle includes steps of 2 h with a 0 nA polarization current and steps of 7 h with either 20 nA or 40 nA polarization currents. The overpotential (calculated from Equation (1)) linked to the gold electrode is null when $i_{pol}=0\;nA$. Then, after application of 20 nA or 40 nA polarization currents, an important evolution of the overpotential is observed (for example, at 500 °C in Figure 7). When $i_{pol}$ is brought back to 0 nA, the overpotential goes back to a value close to 0 V in the same way. The $\alpha_{base}$ coefficient is the only parameter that can increase the difference of overpotential value between a sensor exposed at the same O_2_ concentration for two values of polarization current. The $\gamma_{base}$ coefficient will mainly have an effect on the difference of overpotential value between a sensor exposed at different O_2_ concentrations for the same value of polarization current. The ${k_{0}}_{base}$ value will have an overall effect on the overpotential signal that will be entirely shifted up or down according to the ${k_{0}}_{base}$ value.

Parameters enabling the best fitting results are listed in Table 1 for the two sensors tested. For $\alpha_{base}$ and $\gamma_{base}$, it seems that there is no influence of temperature, whereas for $k_{0_{base}}$, the tendency is clearly an increase of this parameter according to the temperature. With $k_{0}$ being a reduction-constant rate, it seems logical, according to Arrhenius’ law, that its value is increased when the temperature is raised.

**Figure 8:**
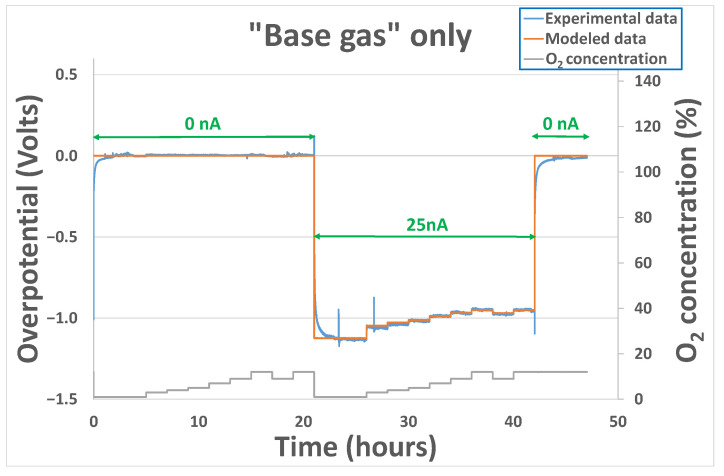
Experimental and modeled overpotential evolution according to the gaseous environment (base gas–O_2_ variation from 1 to 12%) and polarization current sequence (0 nA–25 nA–0 nA) at 500 °C.

Since those parameters are strongly correlated to the kinetics of the reactions occurring at the triple phase boundaries around the gold cathode, the electrode history and the “chemical state” of this last one at the beginning of the experiment (species that remains sorbed, chemical availability of the triple phase boundaries) will play a non-negligible role in the sensor’s behavior. Therefore, another test was performed on a sensor coming from another batch of production. Overpotential evolution results, shown in Figure 8, were obtained for the sensor heated at 500 °C and exposed to base gas only, with different oxygen concentrations varying from 1% to 12%. No polarization current was applied from the beginning to 21 h. Then, after the application of a 25 nA polarization current, an important decrease in the overpotential is observed in the same way as previously. After 42 h, polarization is brought back to 0 nA, and the overpotential goes back, as previously, to a value close to 0 V. Initial values (before fitting operation) of the $k_{0_{base}}$, $\alpha_{base}$ and $\gamma_{base}$ parameters were those obtained with sensor 1 at 500 °C. After fitting, parameter values obtained were coherent with the ones obtained previously: $k_{0_{base}}=3.7\times 10^{-9}$, $\alpha_{base}=0.025$ and $\gamma_{base}=0.13$.

For all the tests performed, the variations of experimental overpotential with O_2_ concentration are well reproduced by modeled data. Nevertheless, it can be noticed that for polarization current transitions from 0 to 20/25/40 nA or from 20/25/40 nA to 0 nA, the fitting error performed is much higher. This can be explained by the fact that, as mentioned earlier, the capacitive part of the phenomenon has not been taken into account in the model. Physically, this capacitance can be linked to the oxygen species adsorbed at the interface electrode-electrolyte-air. In fact, a modification of the polarization current will induce a modification of the sorption equilibrium of O_2_ molecules. As can be seen in Figure 7 and Figure 8, the time required to reach the new equilibrium conditions (steady state) is quite long: 2 h. This can be explained partly by the important volume of the cells in which the sensors are placed during the exposition but mainly by the sorption kinetics, i.e., the kinetics linked to the creation/destruction of bonds between O_2_ molecules and triples phase boundaries around the gold electrode.

### 3.2. Introduction of Pollutant Gases Together with “Base Gas”

While introducing pollutant gases, the variability in the operating conditions becomes important (polarization current, temperature, concentration of oxygen, concentration of pollutant gases)… This section will be divided into two parts and will involve tests operated on different conditions on three other sensors. In the first part, the effect of polarization voltage on the sensors’ output will be studied according to the concentration of pollutant gases (both reductant and oxidant) at a fixed temperature of 500 °C. The third part will be dedicated to the fitting of sensors’ output curves in which the polarization current and temperature remain constant (25 nA and 450 °C, respectively), and the concentration of pollutant gases will be varied together with the concentration of oxygen. Moreover, in each case, the initialization of the “base gas” parameters will be conducted according to the results obtained in Section 3.1.

#### 3.2.1. Effect of Polarization Current

Changing the polarization current can be interesting since measurements performed at different levels of polarization currents could bring a bigger quantity of information exploitable in a view to reach selective detection. Tests have been performed for polarization currents of 20 nA, 50 nA, and 150 nA (to reach overpotential values between 0 V and −1.5 V) on two sensors. Concerning the operating conditions, O_2_ concentration has been set at 12% while pollutant analytes concentrations (NO, NO_2_, and CO) have been varied from 100 ppm to 1000 ppm.

**Figure 9:**
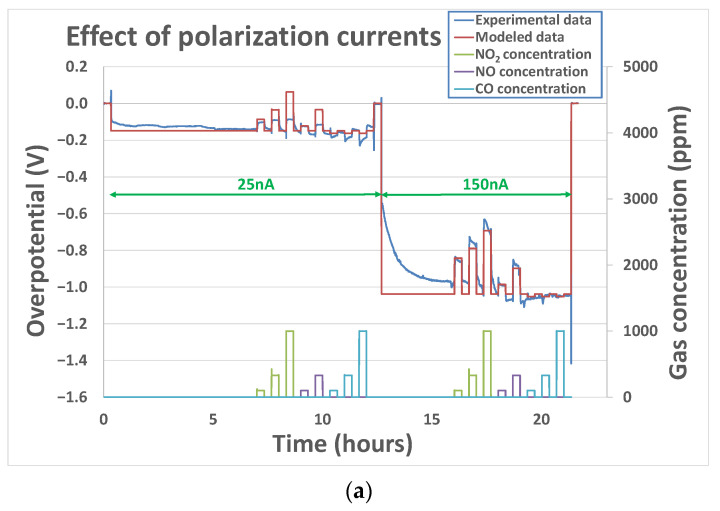

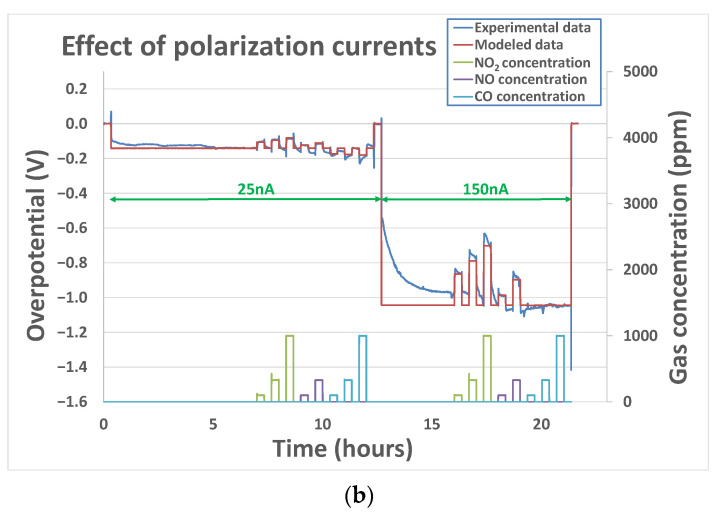
Experimental and modeled overpotential evolution according to the gaseous environment (12% O_2_ + gas concentrations between 100 and 1000 ppm) and polarization current sequence (0 nA–25 nA–150 nA–0 nA) at 500 °C (**a**) with the assumption that $k_{0_{gas}}$, $\gamma_{gas}$ do not change while changing the polarization current; (**b**) with the assumption that $k_{0_{gas}}$, $\gamma_{gas}$ can change while changing the polarization current.

In contrast to the case with base gas only, for which no clear influence of polarization was observed, the couple $k_{0_{gas}}$, $\gamma_{gas}$ seems to be different according to polarization voltage for the tested pollutant analytes. This means that the polarization voltage influences the order of reactions and constant rates. Indeed, as can be seen from the example of Figure 9a), the parameters that have been adjusted for the polarization current of 150 nA are not relevant for a polarization voltage of 25 nA. Indeed, they do not enable a correct fitting of the sensor’s output when the analyte concentrations are varied. Besides, the charge transfer coefficient $\alpha_{base}$, which is, by definition, not supposed to vary with polarization current, has been considered fixed for one analyte. A correct fitting of the experimental curve (Figure 9b)) was obtained while allowing the couple $k_{0_{gas}}$ and $\gamma_{gas}$ to vary according to the polarization current. Parameter-fitting results obtained for the three tested sensors at polarization currents of 25 nA, 50 nA, and 150 nA have been summarized in Table 2 for NO_2_, NO, and CO analytes.

From Table 2, a clear distinction between oxidant gases (NO and NO_2_) parameters and the reductant gas (CO) can be established. It seems that constant rates $k_{0_{gas}}$ and reaction order $\gamma_{gas}$ of oxidant gases are clearly influenced by the polarization current. Overall, an increase in the constant rate and the reaction order has been noticed in the case of oxidant gases. In the case of the tested reductant gas CO, the opposite behavior has been observed. Indeed, when the current reaches 50 nA, the reaction rate becomes so low that no overpotential variation is observed, which is in line with the observations made by Viricelle et al. on equivalent sensors [28], showing that distinction could be made between oxidant and reducing gases variating the polarization current. Therefore, in the case of the CO analyte, it was not possible to extract a constant rate, charge transfer coefficient, or reaction rate.

#### 3.2.2. Effect of Oxygen Concentration

**Figure 10:**
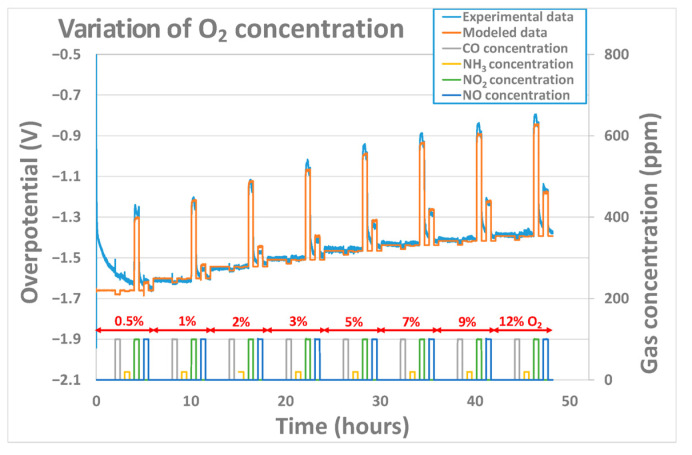
Experimental and modeled overpotential evolution according to the gaseous environment (base gas, CO, NH_3_, NO, and NO_2_) and oxygen concentrations (0.5%, 1%, 2%, 3%, 5%, 7%, 9%, and 12%) at 450 °C.

When used in open-air conditions, the oxygen concentration is not supposed to vary. However, for some applications, like process control in the chemical industry, for example, it can be interesting to validate the model in conditions where the oxygen concentration is not fixed. The robustness of the model was evaluated at 450 °C in conditions where the polarization current is kept constant (25 nA), and the concentration of oxygen is varied from 0.5% to 12% while pollutant concentrations of 100 ppm for NO_2_, NO, and CO and 20 ppm for NH_3_ were introduced periodically and for each O_2_ concentration value. The fitting results obtained were good, as can be observed in Figure 10. Yet, in this case, the fitting parameters extracted (Table 3) for the pollutant gases are more dispersed for the tested sensors than the ones extracted in Table 2, especially for $\alpha_{gas}$ coefficient of oxidant gases (NO and NO_2′_s). Values from 0.003 to 0.015 for $\alpha_{{NO}_{2}}$ and from 0.003 to 0.05 for $\alpha_{NO}$ were obtained. Indeed, those ones vary a lot from one sensor to another and are not comparable with the ones observed in the previous part, whereas the constant rates $k_{0_{gas}}$ and $\gamma_{gas}$ obtained are in the same range of order as the ones observed in the previous part. Additionally, extracted values for NO and NO_2′_s cathodic charge transfer coefficient are significantly lower than the ones obtained in the previous part. This means that the model is not able to describe the evolution of the overpotential of a sensor for which both the oxygen concentration and the polarization current are varied. The cathodic charge transfer coefficient is linked to the velocity at which the electrons are transferred at triple point boundaries, i.e., to the reaction rate. A lower value of the charge transfer coefficient will result in a poor correlation between the electrode potential and the reaction rate. Therefore, it is strongly linked to the surface state of the gold electrode (species adsorbed and roughness), which can vary a lot from one sensor to another and according to the history of one sensor. Then, one hypothesis that can explain the great variability of $\alpha_{NO}$ and $\alpha_{{NO}_{2}}$ between different sensors, while changing the O_2_ concentration, is the fact that the ratio of adsorbed NO_2_ molecules compared to adsorbed O_2_ molecules can be quite different from one sensor to another.

Concerning the reducing gases, the results obtained are consistent with the ones obtained in the previous part. Moreover, it seems that the reaction rate, in the case of NH_3_, is so low that, as for the last part, the three parameters could not clearly be extracted.

## 4. Conclusions

An analytic model of an electrochemical YSZ-based sensor used in polarization mode and exposed to different gaseous mixtures was performed. The model, based on Butler–Volmer equations, was developed and compared to experimental results obtained under different conditions. It relies on two parallel reactions occurring at the gold cathode. The first one is the reduction of O_2,_ and the second one is either the reduction of an oxidizing gas (NO or NO_2_) or the reaction between a reducing gas and O^2−^ anions resulting from the reduction of O_2_. In a view to test the robustness of the model in conditions in which it could be used, the influence of the polarization current and oxygen concentration was studied. The main result concerning the influence of polarization current is that the constant rate of pollutant gases and their reaction order rely on its value. Moreover, for reducing gases, an increase in polarization current will decrease the reaction rate in a way that over 50 nA, the overpotential response is brought to zero for those reducing gases. Additionally, extracted $\alpha_{gas}$ parameters from modeling of tests performed under increasing concentrations of oxygen were more dispersed and different from the ones obtained with a constant concentration of oxygen and a polarization current variating. This means that the model is not suitable for use under experimental conditions where both oxygen concentration and polarization current are varied. The other conclusion for the modeling tests under different concentrations of oxygen was that the extracted parameters were not exportable from one sensor to another, especially concerning the value of the cathodic charge transfer parameter.

The next target for the developed model is to use it in multivariate analysis to reach the objective of both selective and quantitative detection. For that purpose, we need to make sure that the modeling parameters extracted for one sensor in a particular experimental condition (temperature and polarization current) remain constant over time. Therefore, reproducibility tests should be performed and, eventually, a study of the influence of sensor aging on the modeling parameters. Finally, based on the model, the prediction of a simple gaseous mixture composed of a base gas and one of the pollutant gases tested in this work is aimed.

## Figures and Tables

**Table 1:** $k_{0_{base}}$, $\alpha_{base}$ and $\gamma_{base}$ extracted values for temperatures between 450 and 550 °C.

Sensor	Temperature (°C)	$k_{0_{base}}\left(\times 10^{-9}\right)$	$\alpha_{base}$	$\gamma_{base}$
1	450	3.2	0.026	0.09
	500	11	0.025	0.13
	550	25	0.027	0.13
2	450	3.5	0.025	0.10
	500	5.5	0.034	0.10
	550	14.8	0.027	0.09

**Table 2:** $k_{0_{base}}$, $\alpha_{base}$ and $\gamma_{base}$ extracted values for the polarization currents tested at 500 °C.

Gas	Polarization Current (nA)	$k_{0_{base}}\left(\times 10^{-9}\right)$	$\alpha_{base}$	$\gamma_{base}$
Base gas	25–50–150	5–12	0.03–0.04	0.1–0.15
${NO}_{2}$	25	3–5	0.03–0.04	0.25–0.3
	50	30–70	0.03–0.04	0.4–0.6
	150	30–130	0.03–0.04	0.4–0.7
NO	25	4–8	0.06–0.08	0.5
	50	70–170	0.06–0.08	0.5–0.7
	150	120–400	0.06–0.08	0.5–0.8
CO	25	7–25	0.03–0.04	0.09
	50	<0.1	Not assessable	Not assessable
	150	<0.1	Not assessable	Not assessable

**Table 3:** Extracted parameters while varying the oxygen concentration.

Gas		$k_{0_{base}}\left(\times 10^{-9}\right)$	$\alpha_{base}$	$\gamma_{base}$
Base gas		1.5–3.5	0.02–0.035	0.09–0.16
Oxidant gas	NO_2_	60–100	0.0014–0.015	0.3–0.4
	NO	50–470	0.003–0.05	0.4–0.5
Reductant gas	CO	2–7	0.02–0.035	0.09–0.16
	NH_3_	<0.1	Not assessable	Not assessable

## Data Availability

Data are contained within the article.
